# Health Tract No. 92

**Published:** 1865

**Authors:** 


HEALTH TRACT NO. 92.

### REARING CHILDREN.

1. On entering the fourth year, children should not be allowed to eat oftener than once in four hours, but always in peace and cheerfulness.

2. Do not send a child to school, nor allow him to learn at home any thing more than the alphabet, nor commit any thing to memory, except the Lord's Prayer and a half-dozen short, simple, religious hymns, until the sixth year is completed, unless the child will have to "do something for a living" very early.

3. Allow nothing whatever to be eaten within two hours of bed-time.

4. The last meal of the day should be of cold bread and butter, with some mild, warm drink — say milk and water, half and half, sweetened, called "cambric tea"—or a bowl of bread and milk, or mush and milk, made of Indian (corn) or oat-meal. "Preserves," "cake," or other sweet-meats, are most pernicious.

5. Children should sleep in separate beds, on a straw or hair mattress, without caps, being careful to have the feet well warmed by the fire, stockings off; or if in summer, rubbed dry with the hand, washing them every other night. Have extra covering on the feet in cold weather.

6. Encourage them in every way; compel them, if necessary, to be out of doors, or in a large, clean, open, dry, cheerful room, for the greater part of daylight between breakfast and sundown. If the weather is damp or raw, especially at the close of the day, keep them in-doors. In late autumn, winter, and early spring, a child under ten ought not to be out later than an hour before sundown, except in constant, active motion; nine tenths of the cases of croup would be thus prevented.

7. If a child eats at regular hours, do not limit it, except at supper-time.

8. By all means let the child take the fullest amount of sleep. Never wake up a child, except in a day-nap; but be particular to have it go to bed at so early an hour regularly, that it shall wake up of itself in full time to dress for breakfast. Children, left to themselves, are never ready to go to bed, or to get up, in time.

9. Avoid the barbarism of keeping your child still, as long as it is doing no injury to property, person, or good morals. Motion of some sort is a physical necessity to young children; it is an unappeasable instinct. To repress it, by arbitrary commands, is a rebellion against nature and a cruelty to the child.

10. Never threaten a child. It is cruel, unjust, and dangerous. What you have to do, do it, and there make an end; but act deliberately, firmly, kindly, maintaining your own self-respect.

11. Never reprove a child in the presence of any third party; its self-esteem is wounded thereby, and a spirit of self-defense, of opposition, or even defiance is engendered.

12. Never make a positive promise to a child, unless you are perfectly certain you will be able to fulfill it.

13. Always give your child an affectionate greeting on coming home, even after a few hours' absence. It might have been brought to your door a corpse!

14. The most certain and most speedy method of ruining a child is to be forever laying down rules, regulations, and restrictions. At the earliest possible moment it will break away from all restraint.

15. Let nothing ever prevent you from sending your child to bed in a calm and loving and grateful frame of mind. It or you may die before the morning.

16. Be yourself all that you would have your child to be.

